# The Effectiveness of Exercise Interventions Supported by Telerehabilitation For Recently Hospitalized Adult Medical Patients: A Systematic Review

**DOI:** 10.5195/ijt.2021.6356

**Published:** 2021-12-16

**Authors:** Simone Leslie, Junmin Tan, Prudence J. McRae, Shaun P. O'leary, Julie A. Adsett

**Affiliations:** 1 Physiotherapy Department, Royal Brisbane and Women's Hospital, Brisbane, 4029, Australia; 2 School of Health and Rehabilitation Sciences, the University of Queensland, Brisbane, 4072, Australia; 3 Internal Medicine Research Unit, Royal Brisbane and Women's Hospital, Brisbane, 4029, Australia; 4 Physiotherapy Department, Royal Brisbane Aand Women's Hospital, Brisbane, 4029, Australia, School of Health and Rehabilitation Sciences, the University of Queensland, Brisbane, 4072, Australia

**Keywords:** Exercise training, General medicine, Physical therapy, Physiotherapy, Telehealth, Telerehabilitation

## Abstract

**Objective:**

To evaluate the effectiveness of exercise interventions delivered via telerehabilitation (via videoconference) for recently hospitalized adult medical patients.

**Data sources:**

A search was undertaken across six databases for English language publications from inception to May 2020.

**Methods:**

Studies were selected if they included an exercise intervention for recently hospitalized adults, delivered by a physiotherapist via videoconference. Two reviewers independently screened 1,122 articles (21 full text screening) and assessed methodological quality using the Downs and Black Checklist. A narrative synthesis of the included studies was undertaken.

**Results:**

Three studies met eligibility criteria involving 201 participants with chronic heart failure or chronic obstructive pulmonary disease. Findings demonstrated limited evidence supporting the effectiveness of exercise delivered via telerehabilitation in improving physical function and patient reported quality of life outcomes in recently hospitalized medical patients. Telerehabilitation in this setting was also associated with high attendance rates and patient satisfaction.

**Conclusions:**

Findings provide preliminary support for the benefits of exercise interventions delivered via telerehabilitation for recently hospitalized medical patients. Results do need to be interpreted with caution as further high-quality studies specific to this method of exercise intervention delivery are needed.

Functional decline is a common complication of hospitalization for acute medical illness in older people (Covinsky et al., 2003; Dharmarajan et al., 2020; Sager et al., 1996). This can manifest as new impairments in mobility, basic self-care, and instrumental activities, and may persist for months following discharge (Dharmarajan et al., 2020; Sager et al., 1996). For many, referral to exercise-based rehabilitation following an acute medical admission is important for reconditioning and return to higher functional status (Kortebein, 2009). For people admitted to acute medical wards with chronic obstructive pulmonary disease (COPD) or chronic heart failure (CHF), disease specific interventions such as pulmonary and cardiac rehabilitation are recommended to improve exercise capacity, quality of life (QoL) and to minimize the risk of readmission (Anderson et al., 2017; Puhan et al., 2016; Taylor et al., 2014).

Many people are unable to access recommended outpatient rehabilitation services following hospitalization (World Health Organization, 2020). Common barriers include impairments in mobility and function, transport, financial constraints, and availability of programs (Klompstra et al., 2015; Miller et al., 2018; Moschny et al., 2011; Oates et al., 2019). The emergence of the COVID-19 pandemic and transmission risk associated with traditional in-person care has further limited access to outpatient rehabilitation services for many people (Ali & Khoja, 2020). In response to the pandemic, telerehabilitation has emerged as a viable mode of health care delivery facilitated by changes in policy and funding in many countries (Ali & Khoja, 2020; Brennan et al., 2009; Fisk et al., 2020; Parisien et al., 2020; Rizzi et al., 2020). Telerehabilitation encompasses a variety of delivery methods such as telephone, video-conferencing platforms, and virtual reality (Russell, 2007), which improves access to services by allowing instantaneous information exchange (Cottrell et al., 2017). Telerehabilitation (in this review defined as an exercise intervention delivered by a physiotherapist via videoconference) has demonstrated efficacy in various populations including surgical (Moffet et al., 2015; Russell et al., 2011; Sharareh & Schwarzkopf, 2014; Van Egmond et al., 2018), neurological (Amatya et al., 2015; Johansson & Wild, 2011; Tchero et al., 2018) and oncological patients (Larson et al., 2020). Despite this mounting evidence, the delivery of telerehabilitation is yet to be explored in recently hospitalized medical patients.

The purpose of this study was to systematically review the literature pertaining to the benefits of telerehabilitation in recently hospitalized medical patients. Specifically, we sought to describe the physical, functional, and patient-reported outcomes associated with exercise interventions delivered by physiotherapists via videoconference within six weeks of hospital discharge.

## METHODS

### SEARCH STRATEGY AND STUDY SELECTION

The review protocol was registered with the international prospective register of systematic reviews (PROSPERO Registration Number: CRD42020180443). Electronic databases which were searched for relevant studies included PubMed, MEDLINE, CINAHL, PEDro, Embase and Cochrane CENTRAL. All available articles published prior to May 2020 were searched, in addition to grey literature and reference lists from relevant articles. The search terms, listed in Appendix A, included a combination of Medical Subject Headings (MeSH) and appropriate key words.

Studies had to meet the following criteria to be included in the review:

1. Participants: Adults (≥ 18 years) admitted to a medical ward and discharged back to the community.
2. Intervention: Exercise intervention of at least two weeks duration, delivered by a physiotherapist via videoconference within six weeks of hospital discharge.
3. Comparison: Studies were not required to have a comparison group, but potential comparison groups included non-exercise interventions, in-person exercise interventions, or structured exercise interventions delivered via telephone.
4. Outcomes: Any outcome measures for physical or functional performance, such as six-minute walk distance (6MWD), Timed Up and Go (TUG), and muscle strength. Additional outcomes included patient-reported outcome measures, such as level of physical activity, health-related quality of life (QoL), and patient satisfaction. Whilst not a pre-planned outcome, data pertaining to attendance was collected as a process outcome.

Studies were excluded if the participants were less than 18 years old, admitted to hospital due to a neurological, surgical or oncological condition, discharged from a rehabilitation unit, or were discharged to a residential aged-care facility. Case studies, didactic articles, narrative reviews and studies published in a language other than English were also excluded.

Titles and abstracts of the records retrieved through the database searches were independently screened by two authors (SL and JT). Full-text articles were retrieved if further information was required to determine eligibility. Disagreements between authors were resolved via discussion and consensus was reached without the need for arbitration by a third reviewer.

### RISK OF BIAS ASSESSMENT

The quality of all included studies were independently assessed by two reviewers (SL and JT) using the 'Checklist for Measuring Quality' developed by Downs and Black (Downs & Black, 1998). This tool was developed for evaluation of randomized and non-randomized healthcare intervention trials and is scored according to study quality, external validity, study bias, confounding and selection bias, and study power (Downs & Black, 1998). With a maximum score of 28, publications were rated as: excellent (26-28), good (20-25), fair (15-19) and poor (≤14) (Downs & Black, 1998). Disagreements in quality ratings were resolved by discussion, or through arbitration with a third reviewer (JA).

### DATA EXTRACTION

Information extracted from eligible studies included authors and year, study design, sample size, and participant characteristics (including age, gender and their health condition). Intervention data included telerehabilitation protocols, duration of intervention and follow-up, outcome measures and main findings, and comparator data where available. Whilst attendance was not a primary outcome of the study, it was recorded due to its impact on the feasibility of telerehabilitation as a mode to deliver exercise interventions.

### DATA ANALYSIS

A narrative synthesis was undertaken to report data from the included studies. The type and duration of exercise intervention, outcome measures, and results of the included studies were compared. Due to small study numbers and heterogeneity of interventions and outcomes, a meta-analysis was not feasible.

## RESULTS

### INCLUDED STUDIES

**Figure 1:**
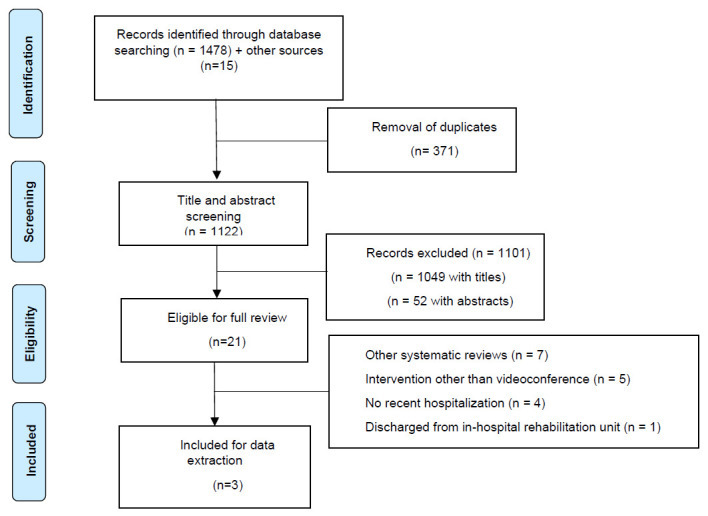
Flow Diagram of the Study Selection Process

The search yielded 1,493 articles, of which 371 were duplicates, leaving 1,122 articles to be screened and excluded. This led to a review of 21 full text articles. Of these, 18 articles were excluded on the basis of: use of a telerehabilitation method other than videoconference; not including an exercise intervention; not including participants recently hospitalized; or articles that were systematic reviews. Figure 1 illustrates the study selection process, resulting in the inclusion of three studies in this review.

Baseline characteristics for the included studies are depicted in Table 1. Two studies recruited participants with CHF (Hwang et al., 2017; Peng et al., 2018) and the other recruited participants with COPD (Minet et al., 2015). The total number of participants across all three studies was 201. They represented an older population with a mean age greater than 65 years. Study locations included Australia (Hwang et al., 2017), Denmark (Minet et al., 2015), and the People's Republic of China (Peng et al., 2018).

**Table 1:** Characteristics of Included Studies

Author	Study design	Number		Mean age, years (SD)		Gender, male n (%)		Primary health condition	Disease severity		
		Exp	Con	Exp	Con	Exp	Con			Exp	Con
**Hwang et al. (2017)**	Randomized controlled trial	24	29	68 (14)	67 (11)	19 (79)	21 (72)	CHF	NYHA n (%) I	3 (13)	2 (7)
									II	9 (37)	21 (72)
									III	12 (50)	6 (21)
**Peng et al. (2018)**	Randomized controlled trial	49	49	<60 29% >60 71%	<60 33% >60 67%	28 (57)	30 (61)	CHF	NYHA n (%) I	11 (22)	13 (26)
									II	18 (37)	18 (37)
									III	20 (41)	18 (37)
**Minet et al. (2015)**	Pre- and posttest intervention study	37	-	69.2 (8.8)	-	5 (14)	-	COPD	FEV1, % (SD)	27.1 (12.5)	-

*Note.* CHF, Chronic Heart Failure; Con, control group; COPD, Chronic Obstructive Pulmonary Disease; Exp, experimental group; FEV1, Forced expiratory volume in 1 second; NYHA, New York Heart Association functional classification.

As demonstrated in Table 2, two studies (Hwang et al., 2017; Peng et al., 2018) were randomized controlled trials specific to patients with CHF. Hwang et al. (2017) compared telerehabilitation to centre-based exercise training, whereas Peng et al. (2018) compared telerehabilitation to a non-exercise control group. The third study (Minet et al., 2015) compared outcomes pre- and post-telerehabilitation for patients with COPD, without a comparator. Exercise programs ranged from three to twelve weeks in length with duration of sessions varying from 30 to 60 minutes. Session frequency ranged from two to five sessions per week. Primary outcome measures were varied and included 6MWD (Hwang et al., 2017; Peng et al., 2018), TUG (Minet et al., 2015), Five Times Sit to Stand Test (FTSST) (Minet et al., 2015), and QoL measures (Minet et al., 2015; Peng et al., 2018).

**Table 2:** Summary of Included Studies

Author	Telerehabilitation-delivered intervention via videoconference	Comparator	Duration of intervention	Follow-up duration	Outcome measures	Main findings	Attendance
**Hwang et al. (2017)**	TR exercise: individualized exercise program, including aerobic and strength training, 60min, 2x/week (videoconferencing to groups of up to 4 participants) Other components: education; additional home exercise program, 3x/week	Centre-based inperson rehabilitation, exercise program similar to TR group	12 weeks	6 months (24 weeks)	6MWD BOOMER 10MWT Grip strength Quadriceps strength MLWHFQ EQ-5D CSQ-8	No significant between-group difference for 6MWD (F_(1.6)_ =1.39, p=0.24) No between-group difference for BOOMER, 10MWT, muscle strength (grip and quadriceps strength) No between-group difference for MLWHFQ and EQ-5D High levels of patient satisfaction reported with no between-group difference	Mean difference (95% CI) of sessions attended was 6 (2–9) in favor of TR
**Peng et al. (2018)**	TR exercise: Stage 1 (weeks 1-4): endurance training (walking and jogging), 40-70% HRR, 20min, 3x/week Stage 2 (weeks 5-8): endurance + resistance training (walking, jogging, calisthenics and strengthening exercises), 40-70% HRR, 30min, 5x/week Other components: education including brochure; regular follow-up via phone or consultations via instant messaging every week with cardiac nurses	Usual care (no exercise) with simple discharge education and regular follow-up at clinic	8 weeks	6 months	6MWD MLWHFQ	Significant improvement in 6MWD in telerehabilitation group. No significant change in 6MWD in control group. (Fb=21.87, p<0.001) Significant improvement in quality of life (MLWHFQ) following telerehabilitation compared to control (Fb=8.27, p=0.005).	
**Minet et al. (2015)**	TR exercise: individualized training including thoracic mobilization exercises, cardio training (60-90% max capacity), strength training (60% 1RM) and breathing exercises, 30-45min, 3x/week Other components: 1-2 TR sessions with occupational therapist; participants were asked to train on non-intervention days	No comparator	3 weeks	-	TUG FTSST CCQ	Significant improvement in TUG (p<0.01) Significant improvement in FTSST (p<0.01) Significant improvement in health status (CCQ) (p=0.04)	

*Note*. 6MWD, 6 Minute Walk Distance; 10MWT, 10 Meter Walk Test; BOOMER, Balance Outcome Measure for Elder Rehabilitation; CCQ, Clinical COPD Questionnaire; CSQ-8, Client Satisfaction Questionnaire; EQ-5D, EuroQoL; FTSST, Five Times Sit to Stand Test; HRR, Heart rate reserve; MLWHFQ, Minnesota Living with Heart Failure Questionnaire; QoL, Quality of Life; TR, Telerehabilitation; TUG, Timed Up and Go.

### RISK OF BIAS

Two studies (Minet et al., 2015; Peng et al., 2018) were rated as 'fair' with respect to this parameter and scored 15 and 18 respectively on the Downs and Black checklist (see Appendix B). Hwang et al. (2017) scored 26 and was rated as 'excellent'. Common issues included an absence of participant blinding (Hwang et al., 2017; Minet et al., 2015; Peng et al., 2018), absence of a blinded assessor (Minet et al., 2015), insufficient information to determine whether the intervention was representative of usual care (Minet et al., 2015; Peng et al., 2018), lack of validation that the sample was representative of population (Hwang et al., 2017; Minet et al., 2015; Peng et al., 2018), methodology of reporting those lost to follow up (Minet et al., 2015; Peng et al., 2018), and insufficient evidence related to study power (Peng et al., 2018).

## SUMMARY OF FINDINGS

### PHYSICAL FUNCTION

Telerehabilitation was associated with improvements in physical function in all three studies. In the study of Hwang et al. (2017), improvements following telerehabilitation were similar to those of a traditional centre-based exercise program with respect to 6MWD, muscle strength, balance, and the 10-Meter Walk Test. Change in 6MWD for the telerehabilitation group was not inferior to that for the control group at 12 weeks (F _(1.6)_ =1.39, p=0.24) and there was no significant between-group difference at the 24-week follow-up. Similar results were reported by Peng et al, (2018) who observed improvements in 6MWD in the telerehabilitation group at six months (although not reaching a clinically meaningful threshold) compared to no exercise controls (Fb=21.87, p<0.001) (Peng et al., 2018). Improvements pre- to post-telerehabilitation in the Minet et al. study were also noted with respect to TUG (p<0.01) and FTSST (p<0.01) performances, although there was no control group in this study (Minet et al., 2015).

### PATIENT-REPORTED OUTCOMES

All studies reported telerehabilitation to be associated with improvements in patient -reported outcomes. In patients with CHF, two studies measured QoL using the Minnesota Living with Heart Failure Questionnaire (Hwang et al., 2017; Peng et al., 2018). In Hwang's study, participants were observed to report significant and sustained improvements in QoL from preprogram to post-program with no between-group differences. When compared to the control group in Peng's study, participants in the telerehabilitation group were much more likely to report sustained improvements in QoL. For patients with COPD, telerehabilitation was associated with significant improvements in health status post program (p=0.04) as measured with the Clinical COPD Questionnaire (CCQ) (Minet et al., 2015).

### ATTENDANCE

Hwang et al. (2017) reported program adherence according to the number of sessions attended, with participants categorized as adherent (>80%), partly adherent (20 to 80%) or non-adherent (<20%). Compared to centre-based rehabilitation, participants in the telerehabilitation group were more likely to be adherent (RR 2.39, 95% CI 1.27 to 4.51) and less likely to be classified as partly adherent (RR 0.46, 95% CI 0.23 to 0.92) (Hwang et al., 2017). No participants in the telerehabilitation group were classified as non-adherent (Hwang et al., 2017).

## DISCUSSION

This is the first systematic review to examine the effectiveness of exercise interventions supported via telerehabilitation in recently hospitalized medical patients. Evidence was limited as only three studies met inclusion criteria. Despite this, collectively these studies provide preliminary evidence to support the efficacy of exercise interventions delivered in this manner to this patient population, with one study reporting outcomes comparable to in-person interventions (Hwang et al., 2017).

The findings of this review are consistent with the growing body of literature in other clinical populations supporting the efficacy of telerehabilitation in the management of patients (Cottrell et al., 2017; Laver et al., 2020; Tchero et al., 2018; Van Egmond et al., 2018). For example, a Cochrane review comparing telerehabilitation with in-person rehabilitation post -stroke, observed similar outcomes with respect to activities of daily living, independence in self-care, domestic life, mobility, balance, and health-related quality of life (Laver et al., 2020). Another systematic review showed telerehabilitation to be feasible and at least as effective as in-person care following orthopaedic, cardiac and oncological surgery (abdominal, thoracic and cervical regions) (Van Egmond et al., 2018). In fact, meta-analyses have demonstrated telerehabilitation to have superior outcomes to in-person care with respect to physical function in response to physiotherapy management in musculoskeletal conditions (Cottrell et al., 2017) and QoL post-surgery (Van Egmond et al., 2018). Specifically, these meta-analyses highlight that telerehabilitation may not only serve as a feasible alternative to in-person rehabilitation but in some circumstances may have some advantages over in-person care.

One advantage of telerehabilitation is that it can provide access to rehabilitation services for people otherwise unable to access care. For example, in a country such as Australia where geography poses a major barrier (Miller et al., 2018; Oates et al., 2019) reducing travel requirements is not only convenient but potentially critical to people with limited mobility and/or access to health professionals (Laver et al., 2020). Additionally, telerehabilitation in the home environment facilitates exercise participation with familiar equipment, permitting integration into daily routines (Van Egmond et al., 2018). In our review, the study by Hwang et al. (2017) supports these reported advantages showing higher adherence to a telerehabilitation protocol compared to centre-based rehabilitation sessions in patients recently hospitalized with CHF.

The COVID-19 pandemic has highlighted the importance of alternate rehabilitation models, and has facilitated wider exposure to telerehabilitation in various patient populations. In response, a rapid uptake of telerehabilitation has been implemented to maintain service delivery (Ali & Khoja, 2020), minimize infection risk and to maximize safety (Fisk et al., 2020). There has been a surge in hospital admissions relating to COVID-19 internationally, and a growing body of literature reports prolonged symptoms associated with deconditioning as well as long-term impacts of the disease (Greenhalgh et al., 2020). The volume of these patients who will likely require rehabilitation, coupled with existing demand, will pose a formidable challenge for rehabilitation services. In the same way that acute clinical services have had to rapidly adapt to the demands imposed by the pandemic, so too have rehabilitation services. Telerehabilitation presents a potential opportunity to increase access to rehabilitation of patients debilitated by COVID-19.

There are several limitations of this review. The low number and heterogeneity of studies that met inclusion criteria limits generalizability. Only one of the three studies was scored as being excellent quality and in the absence of available literature, data were limited to disease-specific (CHF and COPD) rehabilitation programs. Therefore, results may not be representative of all recently hospitalized medical patients. Additionally, the mean age of the participants in all three studies was greater than 65 years, warranting further investigation to confirm efficacy of telerehabilitation in a demographically broader, recentlyhospitalized medical patient population. Well-designed randomized controlled trials and mixed methods approaches are required. Studies should include general medical patients who are not otherwise eligible for disease-specific rehabilitation programs, and should explore economic analysis, physical performance measures, and acceptability of telerehabilitation.

## CONCLUSION

This review provides preliminary evidence that exercise interventions delivered via telerehabilitation for recently hospitalized medical patients is feasible and comparably effective to in-person care. While it is unlikely that telerehabilitation will replace in-person care, it appears to be a promising and complimentary alternative for those unable to access in-person care in the early post hospital phase.

## CLINICAL IMPLICATIONS

- Studies suggest telerehabilitation is feasible and acceptable for recently discharged, adult medical patients.
- The COVID-19 pandemic has highlighted an urgent need for alternative models of delivery for exercise interventions for recently hospitalized patients.
- Further well-conducted RCTs are needed to determine the efficacy of telerehabilitation for this population.

## APPENDIX A SEARCH STRATEGY

**Table:**

Search No.	Search Terms
#S1	“telecare” OR “telerehab” OR “telerehabilitation” OR “telemed*” OR “telehealth” OR “videoconference” OR “internet” OR “e-health”
#S2	“exercise” OR “physical activity” OR “recondition*”
#S3	“physiotherapy” OR “physical therapy”
#S4	“general medicine” OR “hospital*” OR “card*” OR “respir*”
#S5	S1 AND S2 AND S3 AND S4
#S6	“hospital discharge” OR “hospital admission” OR “home care services, hospital-based”
#S7	“pulmonary” OR “lungs” OR “lung” OR “respiratory” OR “cardiology” OR “cardiac”
#S8	S1 AND S2 AND S3 AND S6 AND S7

## APPENDIX B ‘CHECKLIST FOR MEASURING QUALITY’ SCORES FOR INCLUDED PAPERS

**Table:**

	Study Aim	Main Outcome	Eligibility Criteria	Intervention	Baseline Confounders	Main Findings	Random Variability	Adverse Events	Attrition Characteristics	Probability Values	Recruitment Population	Representative Sample	Applicable Intervention	Participant Blinding	Assessor Blinding	Data Dredging	Time Period Adjustment	Statistical Analysis	Intervention Compliance	Valid Outcome Measures	Recruitment Source	Recruitment Time Period	Randomization	Allocation Concealment	Intention-to-treat Analysis	Losses to follow-up	Statistical Power	TOTAL SCORE ( / 28)
Huang et al.	Y	Y	Y	Y	Y	Y	Y	Y	Y	Y	Y	N	Y	N	Y	Y	Y	Y	Y	Y	Y	Y	Y	Y	Y	Y	Y	26
Peng et al.	Y	Y	Y	Y	P	Y	Y	N	N	N	N	?	?	N	Y	Y	Y	Y	?	Y	Y	Y	Y	Y	Y	?	Y	18
Minet et al.	Y	Y	Y	Y	P	Y	Y	Y	Y	N	?	N	N	N	?	Y	Y	Y	?	Y	Y	Y	N	N	N	N	?	15
